# Inhibition of Spinal Ca^2+^-Permeable AMPA Receptors with Dicationic Compounds Alleviates Persistent Inflammatory Pain without Adverse Effects

**DOI:** 10.3389/fncel.2016.00050

**Published:** 2016-02-29

**Authors:** Olga Kopach, Volodymyr Krotov, Julia Goncharenko, Nana Voitenko

**Affiliations:** ^1^Laboratory of Sensory Signaling, Bogomoletz Institute of PhysiologyKyiv, Ukraine; ^2^Laboratory of Synaptic Imaging, Institute of Neurology, University College LondonLondon, UK; ^3^International Center for Molecular Physiology, Bogomoletz Institute of PhysiologyKyiv, Ukraine

**Keywords:** dicationic compounds, Ca^2+^-permeable AMPA receptors, the activity-dependent inhibition, dorsal horn, persistent inflammatory pain, antinociception

## Abstract

Upregulation of Ca^2+^-permeable AMPA receptors (CP-AMPARs) in the dorsal horn (DH) neurons of the spinal cord has been causally linked to the maintenance of persistent inflammatory pain. Therefore, inhibition of CP-AMPARs could potentially alleviate an, otherwise, poorly treatable chronic pain. However, a loss of CP-AMPARs could produce considerable side effects because of the crucial role of CP-AMPARs in synaptic plasticity. Here we have tested whether the inhibition of spinal CP-AMPARs with dicationic compounds, the open-channel antagonists acting in an activity-dependent manner, can relieve inflammatory pain without adverse effects being developed. Dicationic compounds, *N*1-(1-phenylcyclohexyl)pentane-1,5-diaminium bromide (IEM-1925) and 1-trimethylammonio-5-1-adamantane-methyl-ammoniopentane dibromide (IEM-1460) were applied intrathecally (i.t.) as a post-treatment for inflammatory pain in the model of complete Freund’s adjuvant (CFA)-induced long-lasting peripheral inflammation. The capability of dicationic compounds to ameliorate inflammatory pain was tested in rats *in vivo* using the Hargreaves, the von Frey and the open-field tests. Treatment with IEM-1460 or IEM-1925 resulted in profound alleviation of inflammatory pain. The pain relief appeared shortly after compound administration. The effects were concentration-dependent, displaying a high potency of dicationic compounds for alleviation of inflammatory hyperalgesia in the micromolar range, for both acute and long-lasting responses. The period of pain maintenance was shortened following treatment. Treatment with IEM-1460 or IEM-1925 changed neither thermal and mechanical basal sensitivities nor animal locomotion, suggesting that inhibition of CP-AMPARs with dicationic compounds does not give rise to detectable side effects. Thus, the ability of dicationic compounds to alleviate persistent inflammatory pain may provide new routes in the treatment of chronic pain.

## Introduction

Persistent or chronic pain is a prominent healthcare problem worldwide, which is defined by the International Association for the Study of Pain (IASP) as a disease on its own, without apparent biological value that needs to be utterly eliminated after its appearance. Despite considerable efforts, chronic pain remains poorly treatable, attended with side effects and adaptation to a treatment, representing a growing clinical problem that requires new routes based on a mechanism-targeted therapy.

It is known that upregulation of AMPA receptors (AMPARs) in the dorsal horn (DH) neurons causes central sensitization, a specific form of synaptic plasticity in the DH sustainable for a long period of time (Woolf and Salter, 2000; Ji et al., 2003). Peripheral inflammatory pain induces upregulation of Ca^2+^-permeable AMPARs (CP-AMPARs) both at the synapses (Hartmann et al., 2004; Vikman et al., 2008; Park et al., 2009) and the extrasynaptic membranes of DH interneurons (Park et al., 2009; Kopach et al., 2011, 2013), two of those are causally linked to the persistent pain maintenance (Kopach and Voitenko, 2013). Preventing the upregulation of CP-AMPARs in DH interneurons through the interference with molecular mechanisms of AMPAR trafficking has been demonstrated as an effective way to alleviate persistent inflammatory pain at the periphery (Park et al., 2009; Kopach et al., 2013). However, inhibition of central receptors with genetic approaches remains restricted in practical treatment: the preferred focus is the use of conventional compounds.

A substantial variety of blockers inhibiting CP-AMPARs is currently available. They can be divided in two principal groups of: (i) organic toxins (philanthotoxin, joro spider toxin, argiotoxin; Blaschke et al., 1993; Herlitze et al., 1993; Gu et al., 1996) and (ii) dicationic compounds (IEM-1460, IEM-1754, IEM-1925; Magazanik et al., 1997; Tikhonov et al., 2000; Zaitsev et al., 2011). Studies of the effects of organic toxins on pain showed the attenuated development of injury-evoked allodynia when organic toxins (joro spider toxin, philanthotoxin) were used as a pre-treatment (Sorkin et al., 1999, 2001; Pogatzki et al., 2003). However, pain maintenance was unchanged when they were administered as a post-treatment, even in comparatively high doses (Jones and Sorkin, 2004). Strikingly, the effects of dicationic compounds on pain management have not been investigated. In the meantime, they are of special interest since the revealed capability to inhibit CP-AMPARs in the voltage- and the use-dependent manner (Magazanik et al., 1997; Tikhonov et al., 2000; Tikhonova et al., 2008; Zaitsev et al., 2011). The use-dependent inhibition might be the key to impede the development of adverse effects because it “switches off” only functionally active (upregulated) receptors rather than blocking the entire receptor pool. Cumulatively, the effectiveness of use-dependent inhibition correlates with the number of activated receptors/open channels (Zaitsev et al., 2011). Previously, we have demonstrated the increased number of functional CP-AMPARs in DH interneurons in persistent pain conditions: this resulted from promoted insertion of GluR1-containing AMPARs into extrasynaptic plasma membrane (Kopach et al., 2011, 2013) and internalization of GluR2-containing AMPARs from synapses between primary afferents and DH interneurons (Park et al., 2009). In addition, we have shown the capability of dicationic compound IEM-1460 to reverse the inflammatory-induced changes in the AMPAR-mediated currents in DH interneurons (Kopach et al., 2011). Despite the evidenced effects of dicationic compounds at the cellular level, their effects on chronic pain *in vivo* have not been tested yet.

## Materials and Methods

### Animal Care

Animals used in the study were 3–5 weeks-old male Wistar rats. All animal procedures were approved by the local Animal Ethics Committee (Bogomoletz Institute of Physiology, Kyiv, Ukraine) and were performed in accordance with ethical guidelines of the IASP and the European Commission Directive (86/609/EEC). Great care was taken to avoid or minimize any discomfort of the animals with all efforts made to reduce the number of animals used.

### Intrathecal Catheter Implantation

**Figure 1:**
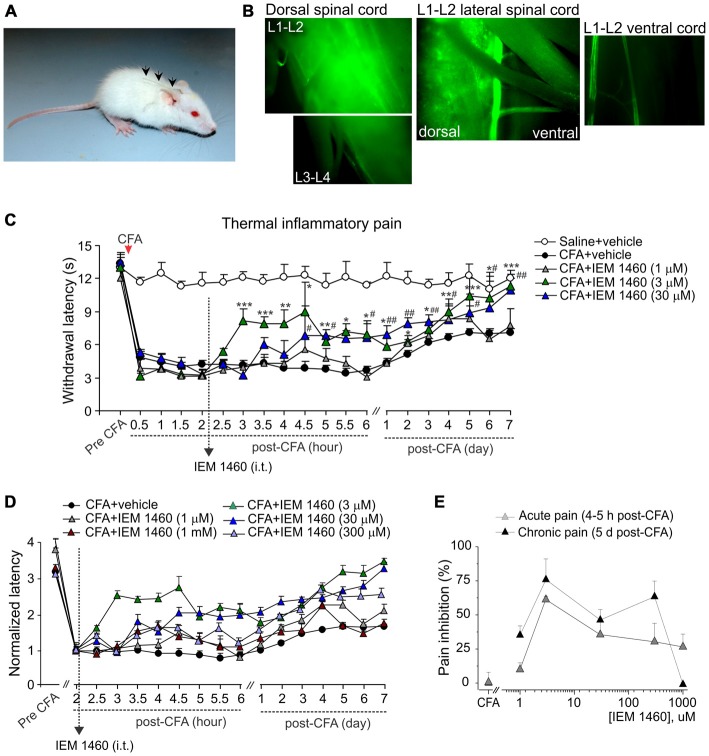
**The concentration-dependent alleviation of thermal nociceptive hypersensitivity by the dicationic compound IEM-1460. (A)** Photograph of a rat with implanted catheter into spinal cord column. **(B)** Fluorescent images of different areas of freshly dissected spinal cord after i.t. injection of morphological dye Alexa-594 (100 μM, 10 μl) into the lumbar spinal cord showing the fluorescence in the dorsal spinal cord (lumbar segments), but not in the ventral spinal cord with notably dim fluorescent signal along the attached roots. **(C)** Hargreaves pain behavioral test in different groups of animals demonstrates that post-treatment of complete Freund’s adjuvant (CFA)-induced inflammatory pain with the dicationic compound IEM-1460 alleviates thermal pain hypersensitivity in rats. Data are expressed as mean ± SEM. *p < 0.05, **p < 0.01, ***p < 0.001 for 3 μM of IEM-1460 vs. the correspondent time-point in the CFA-inflamed group. ^#^*p* < 0.05, ^##^*p* < 0.01 for 30 μM of IEM-1460 vs. the correspondent time-point in the CFA-inflamed group. One-way analysis of variance (ANOVA) and Bonferroni *post hoc* test. **(D)** The paw withdrawal latencies in different groups of animals treated with IEM-1460 were normalized to the correspondent value prior to starting the treatment (2 h) to demonstrate the time-dependence of pain relief on the compound concentration. **(E)** The concentration-inhibition curves of IEM-1460-induced inhibition of inflammatory pain for the acute and the long-lasting responses.

For local delivery of dicationic compounds into the spinal cord we used intrathecal catheter implantation according to a method previously described for rats (Kopach et al., 2013). Briefly, a rat was anesthetized with an intraperitoneal (i.p.) injection of a mixture of ketamine (Farmak, Ukraine) and xylazine (Farmak, Ukraine) in the doses of 70 mg kg^−1^ and 25 mg kg^−1^, respectively. Anesthetized rat was placed in a stereotaxic frame with the head securely fixed between ear bars. One incision was made below the nape 1 cm caudal to the neck. The external occipital crest muscles were retracted with extra care to expose the atlanto-occipital membrane. A polyethylene tube (PE-10) was inserted into the subarachnoid space at the rostral level of the spinal cord region around Th_10_-L_2_ spinal segments through an incision at the atlanto-occipital membrane (Figure 1A). Extra care was taken to avoid any damage of the spinal cord. The incision was closed with silk sutures and treated with Betadine (Gedeon Richter Ltd, Budapest, Hungary). Animals received postoperatively bicillin (0.6 million U kg^−1^, i.p., Farmak, Ukraine) and dexamethazon (60 mg kg^−1^, i.p., Farmak, Ukraine) and were maintained in a warmed area until full recovery from anesthesia. Animals were housed postoperatively in a temperature-controlled environment with food and water *ad libitum*. They were recovered postoperatively for several days before being used in experiments (typically 5 days or until the complete healing of surgical incision if needed). Animals showing any neurological deficits were discarded. The position of the catheter has been confirmed in each animal after termination of an experiment.

### Induction of Peripheral Inflammation

To produce unilateral peripheral inflammation and nociceptive hypersensitivity, 50–100 μl of complete Freund’s adjuvant (CFA, *Mycobacterium tuberculosis*) suspended in an oil-saline (1:1) emulsion was injected subcutaneously into the plantar side of one hind paw of the rats. Saline (0.9%; 100 μl) injection was used as a control.

### Intrathecal Drug Administration

Dicationic compounds were delivered intrathecally (i.t.) into the dorsal spinal cord as confirmed with the Alexa-596-mediated fluorescence detected preferentially at the dorsal lumbar spinal cord (L_1_–L_3_ segments), but not at the ventral lumbar spinal cord or along the attached roots (Figure 1B). Different groups of animals (non-inflamed, CFA-inflamed) received a treatment with saline (control), IEM-1460 or IEM-1925 at indicated concentrations. Injection of a drug (10 μl, diluted in saline) was followed by administration of saline (10 μl) to flush the catheter. Dicationic compounds were used as a post-treatment of peripheral hypersensitivity developed after the induction of peripheral inflammation. After validation that peripheral nociceptive hypersensitivity had profoundly developed following an intraplantar injection of CFA, animals were given a single drug injection (at 2 h post-CFA) or two drug injections (at 2 h and at 5 h after injection of CFA). Timing of starting a treatment was based on our earlier studies of the time-dependent inflammatory changes in the DH GluR2 phosphorylation (Park et al., 2009) and the AMPAR-mediated currents in lamina II DH interneurons (Kopach et al., 2012) in the same model of long-lasting peripheral inflammation in rats.

For assessment of possible side effects of the compounds animals were thoroughly observed for the signs of motor deficit, impaired/irregular movement, stress and anxiety over the entire period of testing an animal. In addition, groups of animals, which received dicationic compounds alone (without induction of peripheral inflammation), were tested for their behavioral responses to peripheral stimulation of different modality (thermal, mechanical) and for sings of possible locomotive deficit and/or the anxiety-like behavior following treatment.

### Behavioral Testing

Behavioral tests were performed in a quiet room, by the experimenter evaluating the animal’s behavior in a blind manner (the experimenter did not know if the animals were treated with saline or drug).

#### Hargreaves Plantar Test

The paw withdrawal responses to thermal stimuli were measured using the Hargreaves technique as described in details previously (Park et al., 2009; Kopach et al., 2013). Briefly, after an animal was habituated to a Plexiglas chamber located above a light box, a radiant heat was applied to the middle of the plantar surface of one hind paw. The intensity of thermal stimulus applied to the plantar skin was adjusted to temperature of ~46°C (30 s cut off). The light beam was automatically turned off when the animal lifted its paw. The time between starting the stimulus and lifting the paw was defined as the paw withdrawal latency. The trial was repeated 3–5 times for each paw with intervals between measurements for at least 3–4 min. For representation of the time-dependent changes produced by dicationic compounds in various concentrations (Figure 1D), the paw withdrawal responses in different experimental groups were normalized to the correspondent value measured prior to starting the treatment (2 h post-CFA) as indicated.

#### Method of von Frey’s Monofilaments

The paw withdrawal responses to the repeated mechanical stimuli were measured using the method of von Frey monofilaments as described previously (Park et al., 2009; Kopach et al., 2013). Briefly, after an animal was habituated to a Plexiglas chamber on an elevated mesh screen (at least 10–15 min prior to testing), the von Frey monofilaments of different intensity of stimulus (Bioseb) were applied to each hind paw. The monofilaments were chosen according to our previous studies of the development and maintenance of the mechanical hypersensitivity and allodynia in the CFA-induced model of peripheral inflammation in rats (Kopach et al., 2012, 2013). The trial was repeated 10 times for each hind paw with an interval between filament applications for at least 1 min. The percentage of responses was calculated for each trial and was defined as the paw withdrawal frequency.

#### Open-Field Test

For assessment of changes in locomotion and general activity of animals in the persistent pain conditions or during an experimental treatment, the open-field test was performed. The open-field test (open-field arena) represents a robust assay for an integrative analysis of animal activity (such as exploratory behavior, sedation, stress/anxiety etc.) as described in detail elsewhere (Bellavance and Beitz, 1996; Bailey and Crawley, 2009). We assessed the animal locomotion and the anxiety-like behavior (which is commonly used to represent side effects) by testing animals in an open-field arena during the defined period of time. For this, a rat was placed in the open-field arena representing a 75 cm × 75 cm × 40 cm wooden box with a digital camera (Logitech C270) attached above a center of the arena to record relocations of animal through the box. Tested animal was allowed to move freely within box during 5 min, often used session duration to sufficiently capture the critical components of animal’ behavior. The total distance traveled by an animal horizontally was analyzed offline and defined as the index of locomotion. The anxiety-like behavior (Buccafusco, 2009) was assessed by the animal movement with regard to the time spent in the box corners, traveling close to the walls (indicating increased anxiety) and crossing the central area (representing the innate behavior of animals to explore novel environments). Testing was typically performed at the same period of time to minimize influencing of animal behavior with a daily activity of laboratory animals.

### Statistical Analysis

All data are presented as mean ± standard error of the mean (SEM) with *n* referring to the number of animals tested. The statistical difference between experimental groups was analyzed by one-way or two-way analysis of variance (ANOVA) followed by Bonferroni *post hoc* test where appropriate. A *p* value of less than 0.05 was considered as statistically significant.

### Experimental Drugs

CFA was purchased from Sigma-Aldrich Company Ltd. (St. Louis, MO, USA and Dorset, UK). 1-trimethylammonio-5-1-adamantane-methyl-ammoniopentane dibromide (IEM-1460) was purchased from Tocris Bioscience (Ellisville, MO, USA). *N*1-(1-phenylcyclohexyl)pentane-1,5-diaminium bromide (IEM-1925) was provided by Prof. L. Magazanik and Dr. D. Tikhonov (I.M. Sechenov Institute of Evolutionary Physiology and Biochemistry RAS, Saint-Petersburg, Russia) and purchased also from Insight Biotechnology (Wembley Middlesex, UK).

## Results

For assessment of a capability of dicationic compounds to alleviate nociceptive hypersensitivity during persistent pain conditions, two different compounds, IEM-1460 and IEM-1925, those high-potent ability to inhibit CP-AMPARs in the use-dependent manner has been proved *in vitro* and *in situ* (Magazanik et al., 1997; Buldakova et al., 1999; Tikhonov et al., 2000; Tikhonova et al., 2008; Zaitsev et al., 2011), were tested *in vivo* in a model of the CFA-induced long-lasting peripheral inflammation. Dicationic compounds were used as a post-treatment of inflammatory pain that had developed after induction of peripheral inflammation. The compounds were administered i.t. at different concentrations to figure out their efficacy in alleviating the inflammatory pain hypersensitivity of different modalities (thermal, mechanical) and in recovering the locomotive deficit and depressed activity of inflamed animals.

### Dicationic Compound IEM-1460 Efficiently Alleviates Inflammatory Hyperalgesia

First we tested whether the dicationic compound IEM-1460 could produce antinociceptive effect on thermal pain hypersensitivity that had developed after the induction of CFA-induced unilateral inflammation in rats. Consistent with our previous studies and those of others (Park et al., 2008, 2009; Kopach et al., 2012, 2013), intraplantar injection of CFA into a rat hind paw produced a robust thermal hypersensitivity on the ipsilateral (but not on the contralateral) side, which developed rapidly (30 min to 1 h after injection), maintained at a peak level for next hours and persisted over several weeks (*n* = 20, *p* < 0.01), representing the period of CFA-induced inflammatory pain maintenance (Figures 1C,D). IEM-1460 given i.t. as a post-treatment of CFA-induced inflammatory pain markedly alleviated the inflammatory-induced thermal hypersensitivity. Relief in inflammatory pain appeared shortly after the compound administration (within 1–2 h after i.t. IEM-1460 application that was 3–4 h post-CFA) and was manifested as the increase in the paw withdrawal latency of inflamed paw, reduced after the induction of inflammation. This increase produced by IEM-1460 reflects the reduction in thermal hyperalgesia; it has been observed within hours after starting the treatment (acute effect) and lasted for at least seven consecutive days after treatment, reflecting the long-lasting antinociceptive effects of the compound (Figures 1C,D). The relief in pain was concentration-dependent, displaying the profound antihyperalgesic effect produced by IEM-1460 at the micromolar concentration range, 3–300 μM, for both acute and chronic responses (Figure 1E). The fastest outcome for pain relief following IEM administration was observed when IEM-1460 was administered at the concentration of 3 μM (Figure 1D), a concentration very close to the estimated IC50 for blocking specifically CP-AMPARs (Buldakova et al., 1999). In particular, the paw withdrawal latency in inflamed-animals, which received 3 μM of IEM-1460 (*n* = 7 per group), has been recovered by approximately 60% of its pre-inflammatory level after 2 h of starting the treatment. After 5 days of treatment with 3 μM IEM-1460, the thermal pain sensitivity of inflamed paw was comparable with those of non-inflamed animals (saline-treated, *n* = 6 per group, *p* > 0.05; Figures 1C–E). Lowering concentration of IEM-1460 to 1 μM or on the contrary increasing it to 1 mM (the estimated IC50 value for blocking GluR2-containing Ca^2+^-impermeable AMPARs; Buldakova et al., 1999) resulted in a short-term reduction in thermal hyperalgesia, observed as a peak increase of the paw withdrawal latency (or a plateau of 1-2 h duration), which steadily declined returning back to the CFA-inflamed level (Figures 1C,D).

**Figure 2:**
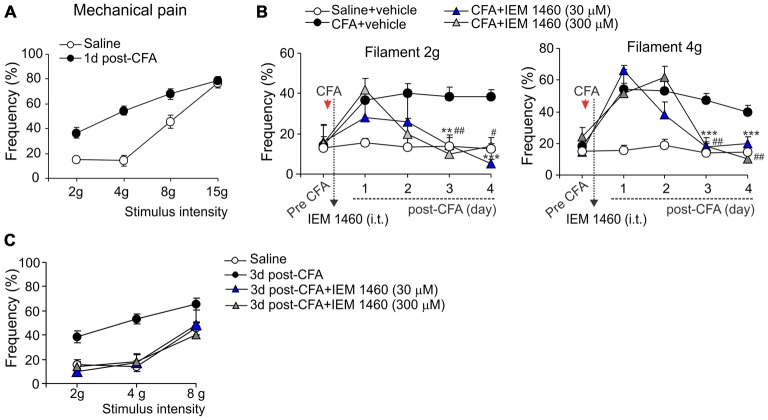
**Analgesic effect of IEM-1460 on the inflammatory-induced mechanical hypersensitivity. (A)** von Frey behavioral test demonstrates tactile allodynia in the CFA-inflamed rats in response to von Frey monofilaments of different stimulus intensity. **(B)** Treatment with IEM-1460 recovered the paw withdrawal frequency in response to the 2 g (left graph) and 4 g (right graph) von Frey monofilaments. Results are presented as mean ± SEM. **p < 0.01, ***p < 0.001 for 30 μM of IEM-1460 vs. the correspondent time-point in the CFA-inflamed group. ^#^*p* < 0.05, ^##^*p* < 0.01 for 300 μM of IEM-1460 vs. the correspondent time-point in the CFA-inflamed group. One-way ANOVA and Bonferroni *post hoc* test. **(C)** Post-treatment with IEM-1460 recovered tactile allodynia in the CFA-inflamed rats after 3 days of treatment.

The capability of dicationic compound IEM-1460 to alleviate persistent inflammatory pain *in vivo* was next tested for the inflammatory-induced mechanical hypersensitivity using the method of von Frey monofilaments. Consistent with previous results (Park et al., 2008, 2009; Kopach et al., 2012, 2013), injection of CFA produced a profound allodynia and the robust mechanical hypersensitivity in response to the von Frey monofilaments of different intensity (Figure 2A) that revealed on the ipsilateral (but not on the contralateral) side. IEM-1460 alleviated the CFA-induced allodynia and mechanical hypersensitivity when applied at a micromole concentration range according with the sufficiently alleviated chronic thermal hypersensitivity, demonstrated above. IEM-1460 at the concentration 30 μM or 300 μM substantially reduced the inflammatory-induced mechanical hypersensitivity in response to the 2 g and 4 g von Frey monofilaments, which produced around 50% of responses in inflamed rats (the mechanical pain threshold), after 2–3 days of treatment (*n* = 5 per group; Figure 2B). After 3 days of treatment, mechanical sensitivity of inflamed paw was indistinguishable from those of non-inflamed animals (*p* > 0.05; Figures 2B,C), indicating that IEM-1460 markedly shortened mechanical pain maintenance. Together, these data demonstrate that dicationic compound IEM-1460 efficiently alleviates inflammatory hyperalgesia of different modalities (thermal, mechanical) and shortens chronic pain maintenance acting within the micromole concentration range.

### The Recovered Locomotion and Diverted Anxiety-Like Behavior of Inflamed Animals After Treatment with IEM-1460

**Figure 3:**
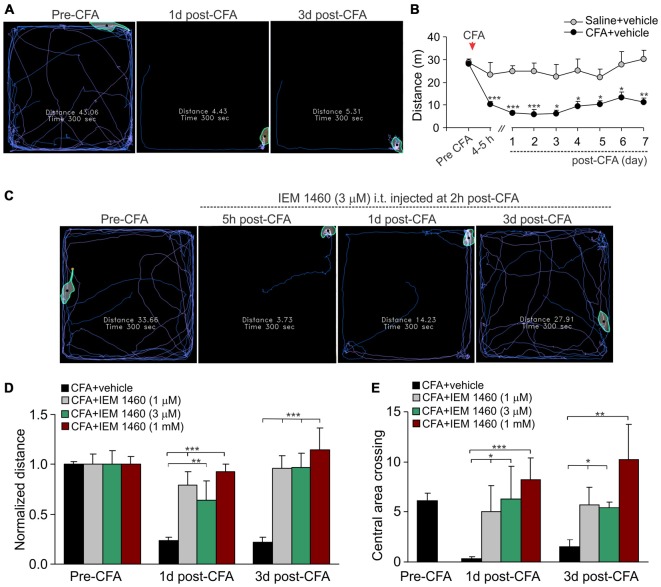
**The IEM-1460-induced relief in locomotive deficit and the anxiety-like behavior of inflamed animals. (A)** The open-field test snapshots demonstrate the severe locomotive deficit in rats with developed peripheral pain syndrome. Sketches of the animal movements taken from the same animal before and at different time-points after induction of inflammation indicating the total distance traveled by an animal for 5 min of recording. **(B)** Summary of the total distance traveled (a 5 min duration) by the saline-treated and the CFA-inflamed animals over the time. Data are expressed as mean ± SEM. *p < 0.05, **p < 0.01, ***p < 0.001 vs. the correspondent time-point in saline. One-way ANOVA and Bonferroni *post hoc* test. **(C–E)** Treatment with IEM-1460 recovered the inflammatory-induced locomotive deficit **(D)** and the anxiety-like behavior **(E)** as seen from the representing open-filed recordings taken from the same animal before and after the induction of peripheral inflammation followed by IEM-1460 treatment **(C)**. *p < 0.05, **p < 0.01, ***p < 0.001 vs. the indicated bar. One-way ANOVA and Bonferroni *post hoc* test.

The open-field test is based on the innate behavior of rodents to explore novel area/surroundings, allowing monitoring and assessment of locomotion, exploratory behavior and general activity of tested animals for integrative analysis of sersorimotor function upon a treatment (Bellavance and Beitz, 1996; Bailey and Crawley, 2009). For assessment of the inflammatory-induced locomotive deficit and the impaired activity of animals in persistent pain conditions, we tested animals prior to the induction of peripheral inflammation and at different time-points following the developed inflammatory pain to compare animal locomotion and explorative behavior between conditions. The robust reduction in animal locomotion together with the profoundly suppressed general activity of animals have been seen shortly after the induction of inflammation (within first few hours), which declined further and maintained at a stably low level for a long period of time (Figures 3A,B; for at least few weeks). For instance, the total distance traveled by an inflamed animal on day 1 after CFA injection was reduced up to ninefold as compared to the distance traveled by the animal prior to the inflammation (*n* = 28 per group, *p* < 0.001; Figure 3B) or to that one in the saline-treated group (*n* = 11 per group, *p* < 0.001; Figure 3B). Typically, the animals with inflamed paw spent a time by predominantly sitting in the arena corners with no attempts to explore surroundings over the entire recording session, displaying the anxiety-like behavior during the persistent pain syndrome (Figures 3A,B).

Further studying a capability of dicationic compound IEM-1460 to alleviate persistent inflammatory pain, we have tested whether the pain relief produced by IEM-1460 would accompany by alleviation in the locomotive deficit developed in inflamed animals. We first verified that the impairments had developed after the induction of inflammation and then tested the animals following treatment once a day until the parameters reached a complete recovery (Figure 3C). Treatment with IEM-1460 improved the locomotion of inflamed animals, revealing the increase in the total distance traveled by an animal with inflamed paw (Figures 3C,D). This improvement has been observed even after 1 day of treatment; it further progressed following next days, showing the restored ability of inflamed animals to travel as much as prior to the induction of inflammation after 3 days of treatment with IEM-1460 at either tested concentration (*n* = 5–6 per group; Figure 3D). This is consistent with the time-course of recovery in the CFA-induced mechanical hypersensitivity by IEM-1460, demonstrated above. The alleviated locomotive deficit was accompanied by facilitated exploratory behavior of inflamed animals that was revealed as their increased crossing of the central area of arena (Figure 3E). Such behavior indicates the recovered general activity of inflamed animals after treatment with IEM-1460 along with the diverted stress and anxiety, which had been developed in persistent pain conditions.

### Assessment of Possible Side Effects Developed by Treatment with IEM-1460

For assessment of possible side effects developed upon inhibition of spinal CP-AMPARs with dicationic compound IEM-1460, animals were tested for their responses to thermal and mechanical peripheral stimulations as well as their locomotive and the anxiety-like behavior before and after the treatment with IEM-1460 at different concentrations.

**Figure 4:**
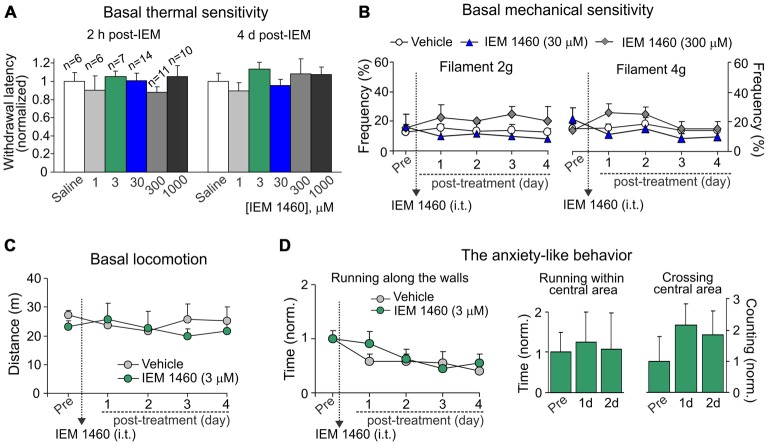
**Dicationic compound IEM-1460 does not give rise to detectable side effects. (A)** The Hargreaves test demonstrates that treatment with IEM-1460 at different concentrations produces neither acute nor delayed changes in thermal peripheral sensitivity. Data are expressed as the mean paw withdrawal latencies normalized to the correspondent time-point in the saline-treated group. **(B)** The von Frey behavioral test shows no changes in the mechanical peripheral sensitivity in response to the 2 g (left graph) and 4 g (right graph) von Frey monofilaments in rats treated with IEM-1460. Results are presented as mean ± SEM. **(C,D)** The open-field test demonstrates no significant changes in animal locomotion **(C)** and the anxiety-like behavior **(D)** after treatment with 3 μM IEM-1460. The anxiety-like behavior was assessed by changes in the time of running along the walls (left graph in **D**), the time spent within the central area of arena (middle bars in **D**) and crossing by animal the arena center (right bars in **D**); results are presented as mean values normalized to the correspondent time-point prior to the treatment (Pre).

To thoroughly evaluate if an acute and/or a delayed adverse effect might develop upon a treatment, we tested the basal thermal nociceptive sensitivity by measuring the paw withdrawal latency before and every next hour (a 6 h-duration) after starting the treatment and then once a day over a week afterwards. Groups of animals, which received IEM-1460 at different concentrations, did not differ in their withdrawal latency at any time-point tested (within hours or days after treatment) as compared to the value prior to starting the treatment or that value in control littermates (saline; *p* > 0.05; Figure 4A). Similar to this, groups of animals did not also differ with regard to their basal mechanical sensitivity following treatment with IEM-1460 (*n* = 5–6 per group, *p* > 0.05; Figure 4B).

Further, no significant changes were observed in the animal locomotion (*n* = 5, *p* > 0.05; Figure 4C) after inhibition of spinal CP-AMPARs with 3 μM IEM-1460, which produced the fastest outcome for modulating pain *in vivo* and also represents the estimated IC50 for blocking CP-AMPARs (Buldakova et al., 1999). Finally, IEM-1460 (3 μM) did not give rise to the anxiety-like behavior, the commonly used readout of side effects, since none of three parameters tested for the signs of increased anxiety were significantly different between the IEM-1460-treated animals and the control (saline-treated) group (*n* = 5 per group for 3 μM IEM-1460, *n* = 11 per group for saline; *p* > 0.05; Figure 4D). We have analyzed: (i) the total time of running along the walls; (ii) time spent within the central area of arena; and (iii) crossing the arena center (Figure 4D). Notably, animals of both tested groups demonstrated promoted attempts to explore the open-field arena over the entire period of testing (for several days) by crossing the central area rather than running along the walls when compared both parameters at different time-points to those ones taken from starting testing (Figure 4D). Together these data demonstrate that treatment with IEM-1460 produces no adverse effects on basal peripheral sensitivity (neither thermal nor mechanical), the locomotive behavior and on none of tested parameters representing the anxiety-like behavior of tested animals.

### Antinociceptive Effects of Dicationic Compound IEM-1925 on Inflammatory Hypersensitivity and Locomotive Deficit

**Figure 5:**
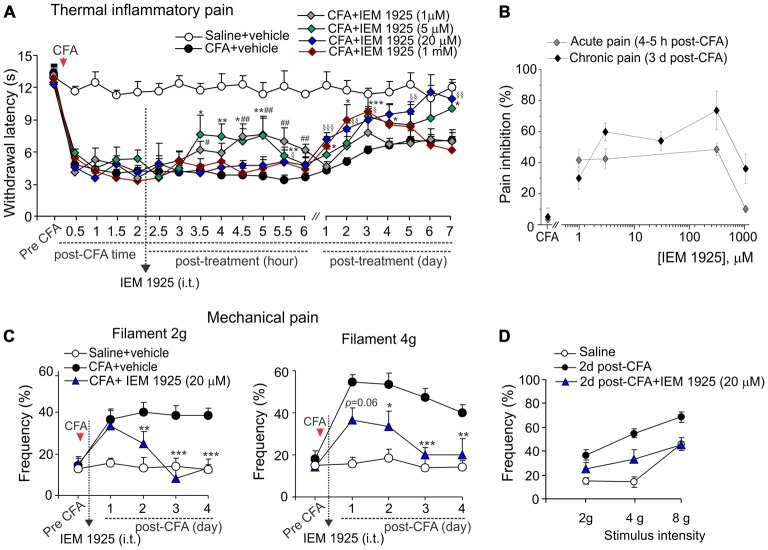
**Antinociception produced by dicationic compound IEM-1925 on the inflammatory-induced thermal and mechanical hypersensitivities. (A)** The Hargreaves pain behavioral test demonstrates the reduced thermal inflammatory hypersensitivity following treatment with IEM-1925 at different concentrations. Data are expressed as mean ± SEM. *p < 0.05, **p < 0.01, ***p < 0.001 for 5 μM of IEM-1925 vs. the correspondent time-point in the CFA-inflamed group. ^#^*p* < 0.05, ^##^*p* < 0.01 for 1 μM of IEM-1925 vs. the correspondent time-point in the CFA-inflamed group. ^§^*p* < 0.05, ^§§^*p* < 0.01, ^§§§^*p* < 0.001 for 20 μM of IEM-1925 vs. the correspondent time-point in the CFA-inflamed group. One-way ANOVA and Bonferroni *post hoc* test. **(B)** The concentration-inhibition curves of the IEM-1925-mediated inhibition of the thermal inflammatory hypersensitivity for the acute and long-lasting inhibitory effects. **(C,D)** The von Frey behavioral test demonstrates the alleviated mechanical hypersensitivity by IEM-1925 (20 μM) in response to the 2 g (left graph) and 4 g (right graph) von Frey filaments **(C)** and the IEM-1925-mediated relief in the inflammatory-induced tactile allodynia in CFA-inflamed animals on day 2 after treatment **(D)**. Results are presented as mean ± SEM. *p < 0.05, **p < 0.01, ***p < 0.001 for 20 μM of IEM-1925 vs. the correspondent time-point in the CFA-inflamed group. One-way ANOVA and Bonferroni *post hoc* test.

Despite the proven high-potent capability of dicationic compound IEM-1925 to inhibit CP-AMPARs in the activity-dependent manner (Tikhonov et al., 2000; Zaitsev et al., 2011), the effects of such inhibition *in vivo* have not been tested yet. Here we have studied a capability of the compound to alleviate thermal and mechanical hypersensitivities and to restore the locomotive deficit and suppressed activity of animals with persistent peripheral inflammation when inhibit spinal CP-AMPARs with dicationic compound IEM-1925 *in vivo*. As in the case of IEM-1460, IEM-1925 markedly alleviated the inflammatory-induced thermal hypersensitivity when used as a post-treatment of CFA-induced inflammatory pain (Figure 5A). The effect of IEM-1925 was dose-dependent, displaying a profound relief in thermal hyperalgesia (either acute or chronic responses) when drug was applied at a micromole concentration range, 5–300 μM (Figure 5B). I.t. administration of IEM-1925 (5–300 μM) resulted in attenuated thermal hypersensitivity, which lasted over the entire period of testing (at least 7 days; *n* = 5 per group for 5 μM IEM-1925; *n* = 15 per group for 20 μM IEM-1925; *n* = 9 per group for 300 μM IEM-1925). Relief in pain produced by IEM-1925 appeared faster than for IEM-1460 used at the similar concentration range. In particular, thermal pain sensitivity of inflamed paw was indistinguishable from that in non-inflamed animals after 3 days of treatment with IEM-1925 (*p* > 0.05; Figure 5A), indicating the profound shortening of the inflammatory pain maintenance by IEM-1925 (Figures 5A,B). Similar to IEM-1460, lowering concentration of IEM-1925 to 1 μM or opposite increasing it to 1 mM (the highest drug concentration tested) produced the reduction of thermal hypersensitivity for a short period of time, which lasted within hours (for 1 μM IEM-1925) or for a few days only (for 1 mM IEM-1925) after treatment with following decline of the paw withdrawal latency back to the CFA-inflamed level (*n* = 6 per both groups; Figure 5A).

IEM-1925 also alleviated the CFA-induced mechanical hypersensitivity and allodynia in inflamed rats. As in the case of thermal hyperalgesia, IEM-1925 alleviated mechanical hypersensitivity faster than IEM-1460. The CFA-induced mechanical hypersensitivity in response to either 2 g or the 4 g von Frey filaments was substantially alleviated even after 2 days of treatment with 20 μM IEM-1925 (*n* = 5 per group; Figures 5C,D); after 3 days of treatment the withdrawal frequency of inflamed paw was comparable to its basal (pre-inflammatory) level (Figure 5C), indicating a complete recovery in peripheral mechanical sensitivity by treatment with IEM-1925.

**Figure 6:**
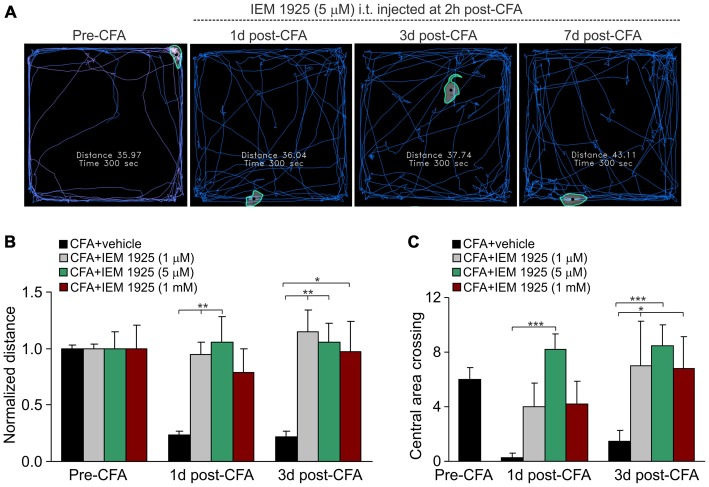
**The IEM-1925-induced relief in locomotive deficit and the anxiety-like behavior in CFA-inflamed animals. (A)** The representative sketches of the open-field test, taken from the same animal, show the distance traveled by the animal before and after the induction of peripheral inflammation, followed by treatment with 5 μM IEM-1925. **(B–C)** Summary of the restored both locomotive deficit **(B)** and suppressed activity of the animals with inflamed paw **(C)** after treatment with IEM-1925. Data are expressed as mean ± SEM. *p < 0.05, **p < 0.01, ***p < 0.001 vs. the indicated bar (the correspondent time-point in CFA-inflamed group without treatment). One-way ANOVA and Bonferroni *post hoc* test.

Finally, the locomotive deficit that had developed in the inflamed animals along to the persistent pain syndrome has been effectively eliminated by treatment with IEM-1925. Again, the outcome in relief produced by the treatment with IEM-1925 revealed faster than that for IEM-1460. In particular, the total distance traveled by inflamed animals was recovered to its pre-inflammatory level even after 1 day of treatment with IEM-1925 at the concentration 1 μM or 5 μM, but not at a high concentration, 1 mM (*n* = 5 per group; Figures 6A,B). This was accompanied by a promoted exploratory behavior of inflamed animals, demonstrating the increased crossing the central area of arena (Figure 6C). The latter indicates that treatment with IEM-1925 declines the anxiety-like behavior in CFA-inflamed animal.

### Dicationic Compound IEM-1925 Affects None of Tested Parameters for Possible Side Effects

**Figure 7:**
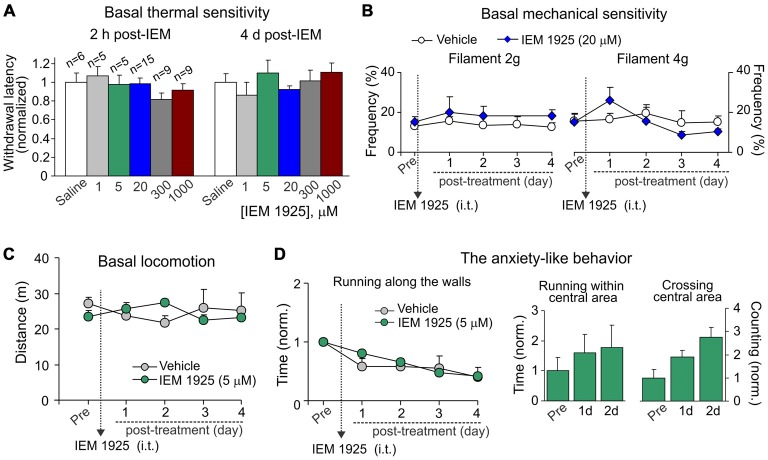
**IEM-1925 does not give rise to detectable side effects. (A)** The Hargreaves test demonstrates neither acute nor delayed changes of thermal peripheral sensitivity in rats after treatment with dicationic compound IEM-1925 at different concentrations. Data are expressed as the mean paw withdrawal latencies normalized to the correspondent time-point in the saline-treated group. **(B)** The von Frey behavioral test shows no changes in the mechanical peripheral sensitivity in response to the 2 g (left graph) and 4 g (right graph) von Frey monofilaments in rats treated with 20 μM IEM-1925. Results are presented as mean ± SEM. **(C,D)** The open-field test shows no changes in animal locomotion **(C)** and no rise in the anxiety-like behavior **(D)** following treatment with 5 μM IEM-1925. Assessment of the anxiety-like behavior was similar as in Figure 4D; results are presented as mean values normalized to the correspondent time-point prior to the treatment (Pre) for all parameters tested.

For assessment of possible side effects developed upon inhibition of spinal CP-AMPARs with dicationic compound IEM-1925, we first tested whether IEM-1925 could influence peripheral sensitivity of animals to different stimulus modalities (thermal, mechanical). No detectable changes in thermal nociceptive sensitivity were found in rats following i.t. administration of IEM-1925 within hours (acute effect) and during next several days after treatment (delayed effect) as compared to their sensitivity prior to starting the treatment or to this sensitivity in the control (saline-treated) group (*p* > 0.05; Figure 7A). Neither of tested concentration of the compound affected the animals’ thermal nociceptive sensitivity over the time. Treatment with IEM-1925 did not also change the basal mechanical sensitivity of animals in response to the 2 g or 4 g von Frey filaments following next several days after treatment (*n* = 6 per group, *p* > 0.05; Figure 7B).

Consistent with these results and those reported above for dicationic compound IEM-1460, IEM-1925 did not change the animal locomotion as determined by the unaltered total distance traveled by an animal following treatment with 5 μM IEM-1925 (*n* = 5 per group, *p* > 0.05; Figure 7C). Treatment with IEM-1925 did not also give rise to the anxiety-like behavior, since the time of running along the walls remained indistinguishable from that in the saline-treated animals (Figure 7D, left graph) and each of tested parameters reflecting the animal exploratory behavior did not decline over the time of treatment with IEM-1925 (Figure 7D, right bars). Thus, IEM-1925 did not give rise to detectable acute and/or delayed side effects in rats.

## Discussion

Cumulative evidence indicates the key role of AMPARs in central sensitization of the DH, a specific form of plasticity in the spinal cord underlying the central mechanism by which peripheral pain develops and is maintained (Woolf and Salter, 2000; Ji et al., 2003). Inhibition of spinal AMPARs with conventional antagonists (NBQX, CNQX, GYKI 52466, CFM-2) reversed mechanical allodynia and both thermal and mechanical hypersensitivities during the development and maintenance of pain of various origins (Sorkin et al., 2001; Nozaki-Taguchi and Yaksh, 2002; Jones and Sorkin, 2004; Park et al., 2008). However, inhibition of AMPARs with competitive antagonists affected the basal peripheral sensitivity and caused sedation (Hao and Xu, 1996; Park et al., 2008) that makes this approach impractical. Studies of the molecular mechanisms of persistent pain maintenance demonstrated the upregulation of CP-AMPARs either at the synapses (Hartmann et al., 2004; Vikman et al., 2008; Park et al., 2009) or at the extrasynaptic membranes of DH interneurons (Park et al., 2009; Kopach et al., 2011, 2013), both of those are causally linked to the maintenance of persistent inflammatory pain, since prevention of the CP-AMPAR upregulation in DH interneurons through targeted genetic interfering with the receptor trafficking machinery has effectively alleviated persistent inflammatory pain at the periphery (Park et al., 2009; Kopach et al., 2013). However, usage of genetic approaches remains restricted in treatment, with preferred focus on commercially available drugs.

Among two principal groups of the selective antagonists of CP-AMPARs, the group of organic toxins has been extensively explored for their effectiveness in managing pain in various pain models (Sorkin et al., 1999, 2001; Pogatzki et al., 2003; Jones and Sorkin, 2004), whereas the effects of dicationic compounds have not been studied *in vivo* at all, leaving the whole class of blockers open for further considerations. In the meantime, a capability of dicationic compounds (IEM-1460, IEM-1754, IEM-1925) to block CP-AMPARs in the activity-dependent manner (Magazanik et al., 1997; Tikhonov et al., 2000; Zaitsev et al., 2011) may provide their potential effectiveness in pain relief. As the open-channel antagonists acting in the use-dependent manner, dicationic compounds inhibit CP-AMPARs only if receptor is open that the effectiveness of blockage correlates with the number of open channels (Zaitsev et al., 2011). Therefore, we have tested two dicationic compounds, IEM-1460 and IEM-1925, which capability to inhibit neuronal CP-AMPARs in the use-dependent manner had been proved already (Magazanik et al., 1997; Tikhonov et al., 2000; Tikhonova et al., 2008; Zaitsev et al., 2011) to figure out their effectiveness in managing persistent inflammatory pain *in vivo*. Dicationic compounds were applied i.t. as a post-treatment of persistent inflammatory pain that had developed in the CFA-induced model of long-lasting peripheral inflammation. Either IEM-1460 or IEM-1925 produced profound alleviation of inflammatory hypersensitivity. Firstly, relief in pain appeared shortly after compound administration (within hours). Secondly, the reduction in inflammatory hypersensitivity lasted for a long period of time, resulting thirdly, in a marked shortening of the period of pain maintenance after treatment with dicationic compounds. The effects obtained with using dicationic compounds here are consistent with those demonstrated previously when we prevented the upregulation of CP-AMPARs in DH interneurons by utilizing a gene-targeted strategy (Park et al., 2009; Kopach et al., 2013). However, the effects of dicationic compounds on inflammatory pain maintenance contrast with those reported for organic toxins (joro spider toxin, philantotoxin), which attenuated the development of thermal injury-evoked mechanical allodynia (Sorkin et al., 1999, 2001), carrageenan-induced hyperalgesia (Sorkin et al., 2001) and mechanical allodynia in the postincision pain model (Pogatzki et al., 2003), but failed to alleviate the pain maintenance (Sorkin et al., 2001; Jones and Sorkin, 2004). Such discrepancy very likely relates to different pain models and reflects also a different involvement of CP-AMPARs in mechanisms underlying development and maintenance of pain of various origins. Consistent with this assumption, the acute effects of dicationic compounds on thermal hypersensitivity were similar to those produced by organic toxins (Sorkin et al., 1999, 2001; Pogatzki et al., 2003).

Dicationic compounds alleviated inflammatory pain in the concentration-dependent manner, displaying the fastest and maximal pain relief when IEM-1460 or IEM-1925 was used at the concentration very close to the value estimated as IC50 for blocking CP-AMPARs (Buldakova et al., 1999). Either compound produced alleviation in persistent inflammatory pain, which: (i) appeared shortly after starting the treatment; (ii) maintained for a long period of time (at least 7 days); and (iii) results in a marked shortening of inflammatory pain maintenance. However, relief in pain produced by IEM-1925 appeared faster than by IEM-1460 (for either thermal or mechanical hypersensitivity) when compared antinociceptive effects between the compounds. Our *in vivo* observation is consistent with the reported higher potency of IEM-1925 to inhibit CP-AMPARs *in vitro* comparing to the potency of other blockers (IEM-1460, IEM-1754) within the group of dicationic compounds (Magazanik et al., 1997; Tikhonov et al., 2000; Zaitsev et al., 2011). Notably, lowering concentration of drug to 1 μM or increasing it to a milimolar range (1 mM, the concentration representing the estimated IC50 value for blocking GluR2-containing Ca^2+^-impermeable AMPARs; Buldakova et al., 1999) resulted in the short-lasted alleviation of inflammatory pain with a negligible effect produced by compounds on the inflammatory pain maintenance. This is in agreement with the causally linked upregulation of CP-AMPARs in the DH interneurons of spinal cord to the persistent inflammatory pain maintenance (Park et al., 2009; Kopach et al., 2011, 2013).

However, a loss of CP-AMPARs may cause adverse effects because of the crucial role of CP-AMPARs in synaptic plasticity that would make the prominent analgesic effects produced by dicationic compounds impractical. Therefore, we have thoroughly tested whether inhibition of spinal CP-AMPARs with dicationic compounds could develop side effects by utilizing a set of different behavioral tests to find out if any one among a set of parameters tested is altered following treatment with dicationic compounds. Our behavioral studies demonstrated that neither peripheral sensitivity of different modalities (thermal, mechanical) nor the animal locomotive behavior was changed following treatment. Further, dicationic compounds did not give rise to side effects developed in tested animals since none of the commonly used parameters representing the anxiety-like behavior had been altered (the time of running along the walls, within the central area of arena, crossing of the arena center). Our *in vivo* results are consistent with the absent changes in basal transmission in the brain *in situ* when dicationic compounds were continuously present (Zaitsev et al., 2011). Cumulatively, it indicates that the activity-dependent inhibition of CP-AMPARs with dicationic compounds does not lead to detectable adverse effects.

Summarizing, dicationic compounds, the open-channel antagonists acting in the activity-dependent manner, effectively relieve persistent pain syndrome by alleviating a repertoire of the inflammatory symptoms, including the peripheral hypersensitivity of different modalities (thermal and mechanical pain), the locomotor deficit and the suppressed activity of injured animals in persistent inflammatory pain conditions. The ability of dicationic compounds to provide relief in chronic inflammatory pain represents a new reliable route for pain management and further perspectives in a mechanism-targeted therapy of chronic pain.

## Author Contributions

OK: research concept; design of experiments; animal surgery and behavioral studies; data analysis and interpretation; drafting and revision of the manuscript. VK: design of experiments; animal surgery and behavioral studies; data analysis and interpretation. JG: design of experiments; animal surgery and behavioral studies; drafting of the manuscript. NV: research concept; design of experiments; supervision of studies; critical revision of the manuscript.

## Funding

This work was supported by the National Academy of Sciences of Ukraine (NASU) Biotechnology, Functional Genomics and DFFD F47/066 Grants to NV, and NASU Grant for Young Scientists to OK.

## Conflict of Interest Statement

The authors declare that the research was conducted in the absence of any commercial or financial relationships that could be construed as a potential conflict of interest.
